# The venom gland transcriptome of the Desert Massasauga Rattlesnake (*Sistrurus catenatus edwardsii*): towards an understanding of venom composition among advanced snakes (Superfamily Colubroidea)

**DOI:** 10.1186/1471-2199-8-115

**Published:** 2007-12-20

**Authors:** Susanta Pahari, Stephen P Mackessy, R Manjunatha Kini

**Affiliations:** 1 Center for Post Graduate Studies, Sri Bhagawan Mahaveer Jain College, 18/3, 9th Main, Jayanagar 3rd Block, Bangalore, India; 2 School of Biological Sciences, University of Northern Colorado, Greeley, CO 80639-0017, USA; 3 Protein Science Laboratory, Department of Biological Sciences, National University of Singapore, Singapore 117543 and Deparment of Biochemistry, Virginia Commonwealth University, Medical college of Virginia, Richmond, VA 23298-0614, USA

## Abstract

**Background:**

Snake venoms are complex mixtures of pharmacologically active proteins and peptides which belong to a small number of superfamilies. Global cataloguing of the venom transcriptome facilitates the identification of new families of toxins as well as helps in understanding the evolution of venom proteomes.

**Results:**

We have constructed a cDNA library of the venom gland of a threatened rattlesnake (a pitviper), *Sistrurus catenatus edwardsii *(Desert Massasauga), and sequenced 576 ESTs. Our results demonstrate a high abundance of serine proteinase and metalloproteinase transcripts, indicating that the disruption of hemostasis is a principle mechanism of action of the venom. In addition to the transcripts encoding common venom proteins, we detected two varieties of low abundance unique transcripts in the library; these encode for three-finger toxins and a novel toxin possibly generated from the fusion of two genes. We also observed polyadenylated ribosomal RNAs in the venom gland library, an interesting preliminary obsevation of this unusual phenomenon in a reptilian system.

**Conclusion:**

The three-finger toxins are characteristic of most elapid venoms but are rare in viperid venoms. We detected several ESTs encoding this group of toxins in this study. We also observed the presence of a transcript encoding a fused protein of two well-characterized toxins (Kunitz/BPTI and Waprins), and this is the first report of this kind of fusion in a snake toxin transcriptome. We propose that these new venom proteins may have ancillary functions for envenomation. The presence of a fused toxin indicates that in addition to gene duplication and accelerated evolution, exon shuffling or transcriptional splicing may also contribute to generating the diversity of toxins and toxin isoforms observed among snake venoms. The detection of low abundance toxins, as observed in this and other studies, indicates a greater compositional similarity of venoms (though potency will differ) among advanced snakes than has been previously recognized.

## Background

The advanced snakes (superfamily Colubroidea) consist of a monophyletic group of four families: Atractaspididae, "Colubridae", Elapidae and Viperidae [1]. These snakes have evolved biochemical weapon (toxins), rather than mechanical means of handling prey. Phylogenetic studies show that the venom gland (where toxins are produced) evolved once at the base of the Colubroidea about 60–80 million years ago and has undergone extensive "evolutionary tinkering" of delivery systems and compositions of venom [2,3]. Phylogenetic reconstruction between toxin genes and snake families showed that the recruitment of toxin families into the venom gland has occurred multiple times by both basal (e.g. metalloproteinases, CRISP, Kunitz-type serine protease inhibitors, NGF) and independent (e.g. PLA_2_, natriuretic peptides) recruitment events [4]. Approximately 26 families of toxins have been catalogued in snake venom proteomes, and several families appear to be specific to a particular family of venomous snakes (Additional file 1). Sarafotoxins are found only in venoms of Atractaspididae; serine proteinases related to blood coagulation factors Xa, cobra venom factor, waprins and AVIT (prokineticin) family peptides appear to be limited to the Elapidae; and vascular endothelial growth factor (VEGF), disintegrins, waglerins, dipeptidyl peptidase IV and crotamine occur primarily in venoms of the Viperidae (Additional file 1). The occurrence, relative abundance and pharmacological potency of various members of these toxin families in venom make envenomation remarkably complex. Envenomation by elapid snakes is usually characterized by rapid neurotoxic complications due to presence of large amounts of postsynaptic neurotoxins [5], while envenomation by viperid snakes evokes complex hemorrhagic, hypotensive and inflammatory effects caused by the actions of numerous serine proteinases, metalloproteinases and C-type lectins (CLP) [6-9]. Effects of envenomation by snakes in the genus *Atractaspis *can include vasoconstriction, resulting in cardiac arrest [10]. Despite overall similarity in clinical symptoms exhibited after envenomation by members of a particular family of snakes, there exists considerable species-specific variation in absolute effects within each group, contributing to the difficulty in assessing and treating envenomated victims.

Previously, identification and characterization of venom components relied primarily on various methods in protein chemistry or on cloning of individual genes. However, neither approach is well-suited to detect toxins that are found in low abundance. Therefore, the apparent absence of a particular family of toxins from venom could be due either to their very low abundance or to the lack of expression in the venom gland. The genes of low abundance toxins are best discovered by the construction of a cDNA library and sequencing of a sizeable number of ESTs. Using this approach, new toxin genes in known families as well as several completely new families of toxins have been discovered, and the spectrum of snake toxin proteome is gradually expanding [11-28]. To search for novel and low abundance toxin genes or new families of toxins, we constructed a cDNA library and sequenced ESTs from the venom gland of *Sistrurus catenatus edwardsii *(Desert Massasauga).

*Sistrurus catenatus *(Massasauga Rattlesnake) is a small pitviper broadly distributed across the North American prairies from Ontario, Canada and New York to extreme southeastern Arizona, with an apparently disjunct population in northern Chihuahua, Mexico [29,30]. One subspecies, *S. c. edwardsii *(Desert Massasauga), occurs primarily in arid and desert grasslands, occasionally occurring in dune formations and desert scrub [31-33]. Populations of *S. catenatus *generally are threatened or declining rangewide, primarily as a result of habitat loss and human encroachment, and therefore endangered species status has been recommended [34,35]. In a systematic study, Holycross and Mackessy [33] showed that among Colorado, Arizona and New Mexico populations of *S. c. edwardsii*, lizards are the major prey, followed by small mammals and centipedes. In the present work, the venom gland has been collected from snakes originating from the Colorado population.

General symptoms of envenomation resulting from many North American pitvipers bite are pain, local tissue effects (progressive edema, erythema and necrosis) with coagulopathy (hypofibrinogenemia and prolongation of prothrombin time) and thrombocytopenia as systemic effects. However, there is no specific report to date in the literature concerning envenomation by *S. c. edwardsii*. Profiling of toxin expression of this threatened snake species will give a global view for the expression of all genres of toxins, including variation in coding/noncoding sequences and their evolutionary trends. The results of this study will also help in the understanding of envenomation processes of rattlesnake bites, which in turn will be important for more effective clinical treatment and antivenom management in cases of snakebite.

## Results and Discussion

**Figure 1:**
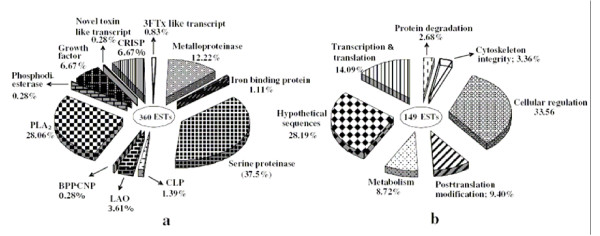
The transcriptome profile of the venom gland of *S. c. edwardsii*. Abundance of (a) toxins and toxin-like transcript clusters, and (b) cellular proteins and hypothetical sequences clusters. Percentage of total ESTs for each category are shown.

A total of 518 out of 576 ESTs produced readable sequences. The sizes of sequences showed a distribution between 300 and 2000 base pairs, with an average of 800 base pairs (data not shown). A total of 232 clusters were obtained and subsequently all clusters checked manually (Table 1; Figures 1a and 1b and Additional files 2 and 3).

**Table 1:** Distribution of 576 ESTs sequenced from *S. c. edwardsii *venom gland in toxin and toxin-like transcript, cellular protein, mitochondrial and hypothetical sequence clusters.

**Transcripts category**	**Number of ESTs**	**Number of clusters**	**Redundancy (clones/cluster)**	**Representation over total clones (%)**	**Representation over matching clones (%)**
Toxin	360	76	4.74	69.40	77
Cellular	107	106	1.04	20.65	23
Mitochondrial	9	9	1.00	1.73	-
Hypothetical	42	42	1.00	8.10	-

The variations observed among toxin isoforms in this library are not due to population level diversity as they are derived from the venom gland of a single snake

A large number of ESTs matched with snake toxins (360 ESTs in 76 clusters; 69.4%). Others code for cellular (non-toxin) proteins (107 ESTs in 106 clusters; 20.65%), and 42 hypothetical ESTs (8.1%). Nine ESTs (1.7%) matched with mitochondrial genes. Fifteen ESTs did not significantly match with any sequence available in non-redundant databases. Further, they do not have any ORFs and may represent either long UTRs (3' or 5') or regulatory RNAs and may have important functions in the rapidly expressing gland tissues. The library contains a large portion of putative toxin genes (69.4%) compared to the cellular EST population (20.6%). We determined the complete sequence of the longest EST of each cluster and sequences were confirmed by repeated sequencing. We completed the sequencing for all major toxins and two low abundant toxin-like transcripts (described below). The existence of genes of particular interest, especially singletons, was confirmed by RT-PCR, using a separate pool of RNA that was used to make cDNA library as template, followed by sequencing.

### Confirmation of species

Taxonomic identification at the molecular level is essential to ensure species identity [36], and 12S and 16S mitochondrial ribosomal RNAs are commonly used in the classification of snakes [37]. Three ESTs for the 12S RNA gene (DQ464268, Additional file 4) in our library show 100% identity to the reported *S. c. edwardsii *ribosomal sequence (AF057227) [38], confirming the venom gland used to make the library is of *S. c. edwardsii *origin. Interestingly, we observed that the ribosomal RNA sequence has poly(A) tail and therefore they appeared in the cDNA library. Polyadenylation of ribosomal RNA has been observed in yeast (*Candida albicans*), fungus (*Saccharomyces cerevisiae*), protistan parasites (*Leishmania braziliensis *and *L. donovani*) and human (*Homo sapiens*) cells, and it is proposed to have a quality control role in rRNA degradation [39-42]. This is a preliminary report showing the possibility of polyadenylation of ribosomal RNA in a reptilian system. On closer examination, we found a putative polyadenylation signal (AATAAA, Additional file 3) [43] sequence six bases upstream of the poly(A) tail.

### Identification of toxin families

#### Serine proteinase

The serine proteinases in the venom gland library of *S. c. edwardsii *are expressed with the highest transcript abundance (38% of 360 ESTs) (Figure 1a) and belong to 19 clusters. Multiple clones appeared in 12 clusters, while 7 were singletons (Additional file 2). One representative EST from each cluster was completely sequenced (DQ464238–DQ464248, DQ439973). One of the clusters (DQ439973) contains only 3'UTR (2 ESTs). This cluster shows 90% similarity with the 3'UTR of a serine proteinase from *Bothrops jararaca *venom gland [44].

Most snake venom serine proteinases (SVSPs) to date are single polypeptide chains, except for two fibrinolytic enzymes from the venom of a Korean Viper, *Agkistrodon blomhoffi brevicaudus *(brevinase, AJ243757 and salmonase, AF176679). In both cases, a single chain precursor is most likely cleaved by proteolysis [45]. In our library, one cluster (DQ464244) shows 90% and 83% sequence identity at the nucleotide and amino acid levels respectively to salmonase. It is not clear whether or not it is also processed to form a heterodimeric serine proteinase in *S. c. edwardsii *venom.

**Figure 2:**
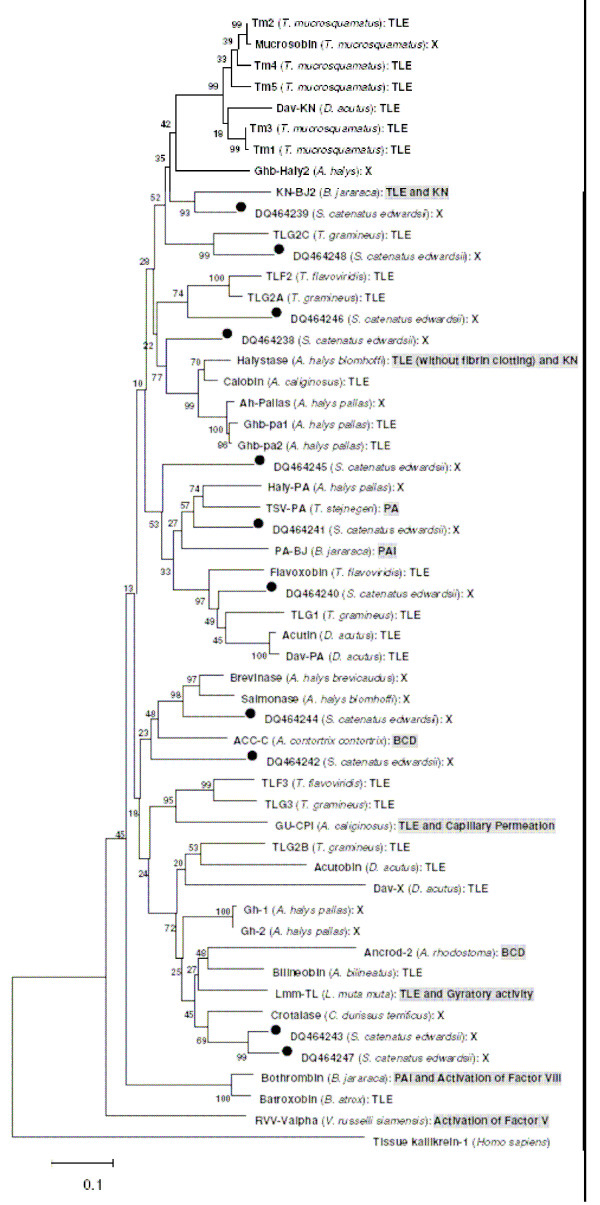
Phylogenetic (NJ) tree of SVSPs. Sequences (complete ORF) available from other pit vipers [47] and 11 isoforms (DQ464238–DQ464248) from this study (filled circle) were used. Tissue kallikrein-1 (P06870) is used as outgroup. The numbers on the branches indicate the bootstrap support values for nodes, and the horizontal bar represents number of substitutions per site. TLE, thrombin-like enzymes; KN, kininogenase; PA, plasminogen activator; PAI, platelet aggregation inducer; BCD, blood clot dispersion; X, activity unknown. Experimentally verified activities are shaded.

The SVSPs are found in all families of snakes and in general, they perturb the hemostatic mechanisms of prey. They act on diverse protein substrates such as fibrinogen, kininogen and platelet receptors [46,47]. Some SVSPs exhibit more than one activity. For example, in addition to their thrombin-like activity, bothrombin, crotalase and LM-TL induce platelet aggregation, kinin release and gyratory activities, respectively [48-50]. We constructed a neighbor-joining (NJ) phylogenetic tree with 11 newly identified SVSP isoforms from *S. c. edwardsii*, to assign putative functions and to examine trends in the evolution of new isoforms [47] (Figure 2). The phylogenetic tree showed a scattered distribution of various isoforms with different pharmacological activities from several species of pitvipers. This pattern indicates that SVSPs diverged after snake lineages speciated. Many SVSPs are commonly considered as thrombin-like enzymes (TLEs) because they mimic the fibrinogenolytic function of thrombin, promoting blood coagulation. Therefore, in most cases only fibrinogenolytic function of SVSPs is tested and the SVSP is categorized as a TLE. However, some thrombin-like enzymes, in addition to releasing fibrinopeptide A and/or B from fibrinogen, also activate protein C [51], complement C3 [52] and platelets [53]. Therefore, it would be interesting to determine the specific pharmacological properties of various SVSP isoforms within each group and map these on their evolutionary relationships.

SVSP genes belong to a multigene family, and the protein-coding regions have been shown to be experiencing accelerated evolution within the venom glands of pitvipers [54]. Such accelerated evolution could lead to the changes in surface loops surrounding the substrate binding site, resulting in the variation of substrate recognition and hence, the function of the protein. The ratio between nonsynonymous and synonymous substitution (*d*_*N*_/*d*_*S*_) of the protein coding sequences of serine proteinase isoforms of this species was found to be 0.99, indicating a trend toward accelerated evolution and therefore divergence in pharmacological function during envenomation.

#### Metalloproteinase and Disintegrin

A total of 44 ESTs fall into 7 clusters and 7 singletons for this family of proteins (12% transcript abundance) (Figure 1a, Additional file 2). One representative EST from each cluster was sequenced (DQ464249–DQ464255). Snake venom metalloproteinase (SVMP) precursors are classified into four groups according to size and domain composition: PI (metalloproteinase domain only); PII (metalloproteinase and disintegrin domains); PIII (metalloproteinase, disintegrin and cysteine-rich domains); and PIV (PIII type domains linked to a lectin-like domain by disulfide bonds) [55]. None of the clusters encode PI type SVMPs.

**Figure 3:**
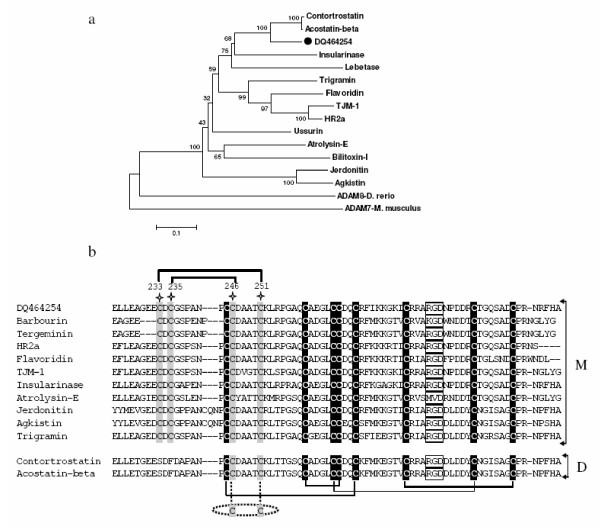
(a) Phylogenetic (NJ) tree for class PII metalloproteinases of viperid venoms. Dataset (complete ORF) used from [60] in addition to one isoform (DQ464254) obtained in this study (filled circle). ADAM8 from *Danio rerio *(Q6PFT3) and ADAM7 (from *Mus musculus*) were used as outgroup. (b) Alignment of the disintegrin domain of class PII SVMPs showing C^233^, C^235^, C^246 ^and C^251 ^(marked in grey) which are proposed to be involved in the formation of both monomeric [M] and dimeric [D] disintegrins in the venom. Only relevant portions of the sequences are shown.

The PII isoform from *S. c. edwardsii *(DQ464254) matches (83–85% identity at the protein level) with the precursors of contortrostatin (Q9IAB0) and acostatin β chain (Q805F6) from *A. contortrix contortrix *venom. Contortrostatin and acostatin are homodimeric and heterodimeric disintegrins, respectively [56,57]. The α chain of acostatin is independently encoded and unlike other disintegrins, it is not derived by proteolytic processing [56]. However, we did not identify any ESTs matching the α chain of acostatin. Phylogenetic analysis shows that DQ464254 is closer to dimeric than monomeric disintegrins (Figure 3a). In the dimeric disintegrins, C^246 ^and C^251 ^form disulfide bridges with the other subunit [58,59]. However, in the monomeric disintegrins, C^233 ^and C^235 ^form disulfide bridges with C^246 ^and C^251^, respectively, making them unavailable for dimerization (Figure 3b). Thus, characteristic Cys residues (C^233 ^and C^235^) are present in all monomeric disintegrins but are absent from dimeric disintegrins [60]. The PII SVMP of *S. c. edwardsii *(DQ464254) contains C^233 ^and C^235 ^in the disintegrin domain and is likely the precursor of a monomeric disintegrin. Other monomeric disintegrins, barbourin and tergeminin, were characterized previously from the venom of *S. miliarius barbouri *and *S. c. tergeminus*, respectively [61].

The main integrin receptor binding motif of disintegrins, RGD, is found to be at the tip of a flexible hairpin loop. Variation of amino acid residues in this motif (R/K/M/W/VGD, MLD, MVD or K/RTS) on the flexible loop confers specificity towards specific receptors, e.g., replacement of R with a K in RGD motif of barbourin and ussuristatin 2 significantly increases the selectivity for α_IIb_β_3 _(fibrinogen receptor) without affecting its binding to α_5_β_1 _(fibronectin receptor) or α_v_β_3 _(vitronectin receptor) [62,63]. Additionally, the residues immediately adjacent to the RGD loop also influence both selectivity and affinity for integrin receptors [64,65]. For example, disintegrins with RGDW and RGDNP have selectively higher affinity for α_IIb_β_3 _and α_V_β_3_, respectively [63]. The RGDNP-containing disintegrins are 10-fold more potent than RGDW-containing disintegrins in blocking the adhesion of cells mediated by α_5_β_1_. The putative disintegrin from *S. c. edwardsii *has RGDNP, compared to RGDW and KGDW in tergeminin and barbourin, respectively. Therefore, further studies of the physiological relevance of variation in receptor selectivity among disintegrins from the same genus will be very informative.

The PIII class of SVMPs are functionally more diverse: they exhibit hemorrhagic activity, inflammatory effects, inhibition of platelet aggregation, apoptosis and prothrombin activation [66-73]. All members of the PIII class of SVMPs have six conserved Cys residues at positions 126, 166, 168, 173, 190 and 206 in their metalloproteinase domain, and some isoforms have a seventh Cys residue at three variable positions (195, 181 or 100) [60,74]. The presence of the seventh Cys residue at position 195 (subgroup PIIIa) results in proteolysis/autolysis, producing a product comprised of the disintegrin-like and cysteine-rich domains (DC domain), whereas when it is present at position 181 (subgroup PIIIb), the formation of a homodimeric structure results [60]). We have not found any isoform having a Cys residue at position 100 (103 in our alignment, Additional file 5) in our library. Two isoforms (DQ464249 and DQ464255) from *S. c. edwardsii *venom possess a seventh Cys residue in positions 195 and 181, and they can be grouped as PIIIa and PIIIb SVMPs, respectively (Additional file 5). Two other isoforms (DQ464250 and DQ464251) do not possess a seventh Cys residue and hence cannot be grouped with any subgroups. Some other isoforms, such as HR1a [75,76] and HF3 [68,77], also do not have the seventh Cys residue in the metalloproteinase domain. We propose that these metalloproteinases be grouped under PIII_0 _(suffix '0' to indicate the absence of the seventh Cys residue) (Additional data file 5). The isoform DQ464253 is a partial segment and it cannot be assigned to any subgroup. However, it shows identity with the A chain of a heterodimeric metalloproteinase identified in the venom of *Vipera lebetina *which induces apoptosis in endothelial cell lines [78]. Overall, the venom of *S. c. edwardsii *appears to have significant molecular variation among metalloproteinases and their derived components.

#### Phospholipase A_2_

Interestingly, in our cDNA library only one cluster of PLA_2 _(DQ464264) was found, despite having the second highest transcript abundance (28%) (Additional file 2, Figure 1a). It matches with an acidic PLA_2_(AAS79430) of *S. c. tergeminus*, with only one amino acid residue (nucleotide) difference at position 80, P(CCG) → Q(CAG), in the mature form. Thus there is no diversity of PLA_2 _in *S. c. edwardsii *venom, though snake venom PLA_2 _is one of the most rapidly evolving enzyme families. In most species, several isoforms of PLA_2 _are observed in cDNA libraries and venoms [79-81], and these have acquired diverse physiological functions [82-84]. This observation is also supported by proteomic analysis of *S. c. edwardsii *venom, while venoms from individuals of other species of *Sistrurus *contain multiple PLA_2 _isoforms [85].

#### Phosphodiesterase

Sequence of a partial singleton EST (transcript abundance 0.28%; Additional file 2, Figure 1a) (DQ464266) shows 60% identity to the C-terminal region of the phosphodiesterase gene from chimpanzee (XP_001168685). This is the first cDNA sequence for a phosphodiesterase from snake venom. Phosphodiesterase activity has been observed in venoms of Elapidae, Viperidae and Colubridae snakes [86-88]; however, the role of this enzyme in envenomation is not yet clear. Venom phosphodiesterases hydrolyze 5'-phosphodiester and pyrophosphate bonds in nucleotides and nucleic acids and release 5'-diphosphates, 5'-monophosphates and purines [89]. Free purines are also present in snake venoms, and they may contribute to envenomation sequelae [90].

#### L-amino acid oxidase

We obtained one cluster having 13 ESTs (transcript abundance 3.5%) (Additional file 2, Figure 1a). The complete sequence (DQ464267) shows high sequence identity (96%) with LAO of *Crotalus adamanteus *venom. LAOs are widely found in snake venoms and in addition to catalyzing the oxidative deamination of amino acids, they affect platelets, induce apoptosis and have hemorrhagic effects [91].

#### C-type lectin

In our library, CLP account for approximately 1.4% abundance and have one cluster (DQ464256) and two singletons (DQ464257 and DQ464258) (Additional file 2, Figure 1a). On BLASTP search, they match with the β subunit of mamushigin (Q9YI92; 80% identity), CHH-B (P81509; 83% identity), and the A chain of Factor IX/Factor X binding protein (IX/X-bp) (2124381A; 86% identity) respectively. Mamushigin, CHH-B and IX/X-bp are heterodimeric; however, in our library, we did not find any match to ESTs encoding the corresponding complementary subunits. Therefore, it may be interesting to examine the CLP-related proteins in this venom and determine their biological properties.

#### Growth factors

We obtained one cluster (transcript abundance 7%) encoding vascular endothelial growth factor (VEGF) (Additional file 2, Figure 1a). Sequencing of 8 clones from this cluster showed there are two isoforms (DQ464259 and DQ464260) with only two amino acid residue (nucleotide) differences at positions 105, Q(CAG) → E(GAG), and 114, K(AAG) → E(GAG). We also sequenced a singleton (DQ464261) encoding nerve growth factor (NGF). Another singleton (DQ464277) matched with the C-terminus of connective tissue growth factor-related protein (CTGF). This is the first report of CTGF-related protein in a venom cDNA library. Its origin in the venom gland, instead of other surrounding tissues, needs to be verified.

#### Cysteine-rich secretory protein

We obtained one cluster (transcript abundance 7%) (Additional file 2, Figure 1a) for a CRISP (DQ464263) which matches with Catrin (AAO62995, 87% identity) from *C. atrox *venom. CRISPs are widely distributed in mammals, reptiles, amphibians, arthropods, nematodes, cone snails and plants, and they exhibit diverse biological functions [92]. They are single chain (MW of ~20–30 kDa), highly conserved proteins organized in three domains: a PR-1 (Pathogenesis Related proteins of group 1) domain, a hinge domain and a cysteine-rich domain (CRD). They contain 16 Cys residues forming eight conserved disulfide bonds. A few snake venom CRISPs have been shown to act upon various ion channels through the CRD domain [93-96]. However, the function of the majority of CRISPs from snake venom is unknown [97]. Therefore, it may be interesting to examine the biological properties of the CRISP found in *S. c. edwardsii *venom.

#### Bradykinin-potentiating peptide and C-type natriuretic peptide

We found a singleton (transcript abundance 0.28%; Additional file 2, Figure 1a) encoding a BPP-CNP (DQ464265) which showed 80% identity with a BPP-CNP precursor from *Lachesis muta *[98]. The BPP-CNP family of proteins lowers the blood pressure of prey during envenomation. Its low abundance in our library indicates that BPP-CNP may not have a significant role in envenomation by *Sistrurus*, unlike bites by other pitvipers (*Bothrops *and *Lachesis*) in Southern America [15,98].

#### Three-finger toxin like transcripts

**Figure 4:**
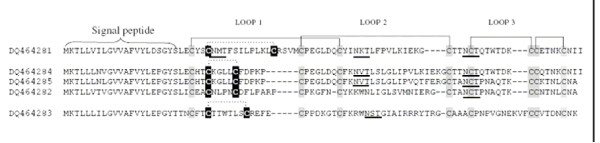
Alignment of amino acid sequences of the putative precursors of 3FTxs. Cys residues which are shaded in grey are commonly present in short chain 3FTx and form 4 disulfide bridges (solid black lines). Cys residues shaded in black possibly form the additional disulfide bridge (dotted black line) present in the non-conventional 3FTx family. Potential N-glycosylation sites are underlined.

We obtained three individual singletons (Additional file 2, Figure 1a) in the library (transcript abundance 0.83%) which belong to the 3FTx family of proteins. As 3FTxs are very uncommon in viperid venoms, using targeted approach we performed RT-PCR using a separate pool of RNA as template and sequenced 96 clones. We found a total of five isoforms of 3FTx-like trancripts (DQ464281, DQ464282, DQ464283, DQ464284 and DQ464285) (Figure 4). They have a signal peptide followed by a mature protein consisting of 64–68 residues. They appear to belong to the non-conventional 3FTxs [99], with five disulfide bridges, and the fifth disulfide bridge is in loop 1 (Figure 4). All isoforms have the potential *N*-glycosylation motif, N-X-T/S (Figure 4).

3FTxs were thought to be found only in elapid/hydrophiid venoms, though the origin of recruitment to the elapid/hydrophid venom proteome is not clear [100]. A polypeptide toxin (8 kDa) which crossreacts with α-bungarotoxin and binds with high affinity to nicotinic acetylcholine receptor (K_d _of 7.3 × 10^-10 ^M in competition with α-bungarotoxin) was isolated from the venom of *A. halys *(a pitviper) [101]. However, no sequence information of this protein is available. Recently, three clones (DY403363, DY403848 and DY403174) were obtained from a cDNA library of *L. muta *venom gland which potentially encode polypeptides similar to 3FTx fold proteins [98]. However, only one clone (DY403363) has the start and stop codons (complete ORF); the other two do not. These sequences do not have any homology, at either the nucleotide or protein levels, to those obtained from *S. c. edwardsii *(this study).

**Figure 5:**
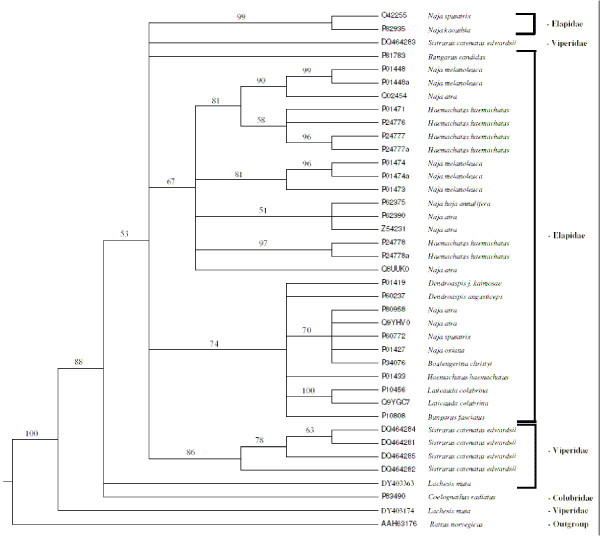
Neighbor-joining cladogram of 3FTx sequences. Numbers preceeding each species name refer to Genbank accession numbers, and numbers before most nodes indicate bootstrap values (1000 replicates).

Phylogenetic analysis of 3FTXs from three families of snakes (Elapidae, Colubridae and Viperidae) was achieved using PAUP 4.10b [102]. Trees obtained using Neighbor Joining (bootstrapping) or Parsimony Analysis (strict consensus, tree is not shown) were somewhat different, but major topological features were retained (Figure 5). One transcript from *S. c. edwardsii *(DQ464283) does not cluster with the other four but falls within a separate clade containing *Naja *and *Bungarus *(both elapids) 3FTxs. Four other transcripts of *S. c. edwardsii *form a monophyletic clade within an exclusively elapid clade. Interestingly, both methods place *L. muta *(a viperid) clones (DY403363 and DY403174) and *Coelognathus radiatus *(a colubrid) 3FTx as basal to all other 3FTxs, suggesting a common origin followed by diversification of 3FTxs among all advanced snakes. Very similar trees, with the same topology of family groups, were obtained using Bayesian analyses and hence support the above conclusions (Additional file 6).

This family of proteins was not observed in a detailed proteomic characterization of *S. catenatus *and *S. miliarius barbouri *venoms [14]. cDNA libraries of other viper venom glands, including *B. jararacussu*, *B. insularis*, *A. acutus *and *Deinagkistrodon acutus*, do not show their presence [15,16,22,26]. This could be due to either low abundance transcripts and proteins or non-uniform recruitment of 3FTx into the venom proteome within Viperidae. In *S. c. edwardsii*, the low transcript abundance (0.83%) suggests that 3FTx are minor components of the mature venom.

In snake venoms, 3FTXs exhibit diverse pharmacological effects due to their ability to target various receptors and ion channels [103]. It is important to note that the β-sheeted loops play crucial roles in binding to various targets, and these regions are the most variable among *S. c. edwardsii *3FTXs. Further, the *d*_*N*_/*d*_*S *_ratio of 0.98 (close to 1) for their coding sequences indicates that a trend towards accelerated evolution is present, as with the serine proteinases. If the variations in the β sheet loop regions are the result of positive selection (accelerated evolution), they may exhibit distinct and novel biological activities.

#### Novel toxin-like transcript

**Figure 6:**
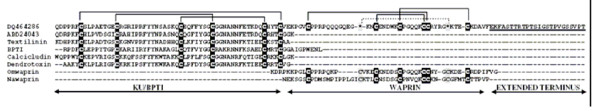
Alignment of the novel toxin-like transcript with snake venom Kunitz/BPTI proteins and waprins. ABD24043 (*Daboia russellii russellii*); Q90W98 (Textilinin, *Pseudonaja textilis textilis*); P81658 (Calcicludin, *Dendroaspis angusticeps*); P00981 (Dendrotoxin-K, *Dendroaspis polylepis polylepis*) and BPTI from Bovine. P83952 (Omwaprin, *Oxyuranus microlepidotus*); P60589 (Nawaprin, *Naja nigricollis*). The presence of conserved disulfide bonds are indicated by solid black lines. The disulfide bond which is missing in the novel toxin but conserved in waprins is indicated by dotted lines. The extended C-terminus of the novel toxin is underlined.

In our library, we obtained one singleton (Additional file 2, Figure 1a) (DQ464286, transcript abundance 0.28%) with an ORF encoding a signal peptide (24 residues) and a mature protein (128 residues). The putative mature protein is rich in Cys residues, similar to many other snake venom toxins. Its N-terminal domain matches with Kunitz/BPTI toxins (53–68% identity) and the middle domain matches with waprins (45–58% identity), and the novel transcript has an extended C-terminus (Figure 6). Both Kunitz/BPTI [104] and waprins [105,106] are found separately as single domain proteins in snake venoms. Two of the Cys residues, which form one of the four disulfide bonds in waprins, are missing in the new transcript (Figure 6). RT-PCR using a fresh RNA (other than used to make cDNA library) as template and sequencing experiments show the presence of this fused transcript in the venom gland and hence it is not an artifact due to template switching by the Reverse Transcriptase used for making the cDNA library [107-109]. Although a number of cDNA sequences of Kunitz/BPTI from snake venoms have been completed, none of them have the waprin domain and the C-terminal extension. Currently, cDNA sequences of waprins are not known. However, this is the first experimental evidence for the presence of a waprin domain (though fused with another toxin) in viperid venom.

The longer ORF having Kunitz/BPTI and waprin domains together could be due to the fusion of two individual genes encoding Kunitz/BPTI and waprin. Gene fusion mediated by exon shuffling (intron mediated recombination or retrotransposition) has been established as an essential genetic mechanism for the origin of new genes in invertebrates, vertebrates and plants [110,111]. Recently, a new genetic process, transcription-induced chimerism (TIC), in cases of tandemly located gene pairs has been shown to be responsible for gene fusion in the human genome, producing chimeric proteins [112,113]. It is not clear at this stage how this novel fused gene has originated in the snake venom gland. This fused transcript may code either for a precursor which is processed to form two individual classes of venom proteins (Kunitz/BPTI and waprin) or a novel toxin with two distinct domains and having a new biological function. It has been observed that new genes often give rise to new biological functions driven by adaptive Darwinian selection [114-116]. The mechanism of fusion of these apparently independent genes, the evolutionary trajectory of this fused gene and the potential new toxic function of the chimeric protein are all areas for future investigation.

#### Iron-binding protein

Four ESTs (Additional file 2, Figure 1a) (dbEST: SCEHYPO1, transcript abundance 1.11%) showed homology with an iron-binding protein with a potential signal peptide. Although most iron-binding proteins are generally categorized as storage protein, some of them, such as ovotransferrin and lactoferrin, have antimicrobial activities [117-119]. It is not clear whether or not this protein is found in the venom. However, omwaprin, a member of the waprin protein family, and the C-terminal region of a myotoxic PLA_2 _were both shown to have antimicrobial activity [105,120].

### Identification of cellular transcripts

We obtained 106 clusters (transcript abundance 21%, 107 sequences) which are involved in various cellular functions, including transcription and translation, secretion, post-translational modification, general metabolism and other functions (Additional file 3, Figure 1b). Similar house-keeping protein products have been observed in other snake venom glands [13,15,22]. One of the ESTs (SCE438) matches (74%) a calcium- and integrin-binding protein which assists platelet spreading [121]. Although modulation of platelet and integrin functions is a key activity of several snake venom components, we do not believe that this protein is present in venom, as it lacks the signal peptide.

Overall, results from our cDNA library demonstrate extensive molecular diversity in the venom composition of *S. c. edwardsii*. Serine proteinase and metalloproteinase isoforms are the most abundant components and in the venom, they exert diverse pharmacological activities, particularly disrupting hemostasis. The numerous minor components likely play an ancillary role in envenomation. These diverse toxin isoforms, together with minor components, may be characteristic of venoms from species utilizing different prey types, such as lizards (ectotherms) and birds and mammals (endotherms) [122].

### Venom composition and genetics of their origin

Snake venoms consist of a diverse range of pharmacologically active protein and peptide toxins which are primarily used in prey capture and secondarily as defense weapons. To date, the majority of the work on toxin identification and characterization has been concentrated on snakes of the families Elapidae and Viperidae because they are often abundant, produce high yields of venom and represent a significant risk to human health worldwide. Recent studies of venom transcriptomes and proteomes indicate that our knowledge of venom composition is partly limited by experimental detectability. For example, 3FTxs, which were thought to be found exclusively in elapid venoms, were detected in viperid venom gland transcriptomes only recently [[98] and in this study]. Similarly, CLP, thought to be limited to viperid venoms, have been detected recently in the venom gland of *Philodryas olfersii *(Colubridae) and *Bungarus *species (Elapidae) [27,106,123,124]. Further, a new family of low abundance toxin (vespryns) was identified in both elapid and viperid venoms [98,123], Therefore, with the application of advanced techniques like EST sequencing, "compositional specificities" between families of venomous snakes may become less distinct (Additional file 1). Multiple recruitment events may lead to an increase in the spectrum of known and unknown toxin families, decreasing the compositional specificities among venomous snakes. However, differential contribution of specific toxins to the overall expressed proteome of venomous snakes does lead to significant differences in venom composition between species.

A central theme in the evolution of venom systems is complete duplication of toxin genes, followed by accelerated evolution which favors nonsynonymous amino acid substitution towards neofunctionalization. Modification of selected surface areas of toxins [82] is responsible for producing the functional diversity in animal (invertebrates: snails and scorpions; vertebrates: snakes) toxin multigene families [125]. However, one important observation in the present report is the occurrence of a novel toxin-like transcript generated by fusion of two individual toxin genes, Kunitz/BPTI and waprin, in a snake venom gland. Though the mechanism for creation of this fused gene needs to be studied further, it clearly indicates that other genetic processes (gene shuffling or TIC) are also operating in the venom gland to create novel toxin genes. Genes originating by other genetic processes such as exon shuffling are recent [111], and therefore the addition of this fused toxin-like transcript to the venom proteome is perhaps new. At this stage, it is tempting to speculate that the origin of modular organization of different classes of SVMPs, which appears to be the result of gene fusion events, may be due to a genetic process other than gene duplication. SVMPs are very abundant toxins and carry out a principal role in envenomation by viperid snakes, and therefore studies of their genetic origin and organization will be of great interest. Circumstantial evidence of trans-splicing for the generation of serine proteinase isoforms in the venom gland of *V. lebetina *has been presented [126]. Kopelman *et al.*[127] have shown that alternative splicing and gene duplication are inversely correlated evolutionary mechanisms. According to Parra et al. [113], only 4–5% of the tandem gene pairs in the human genome can produce chimeric proteins. It is obvious that these alternative genetic processes responsible for expanding functional proteomes are uncommon among biological systems, and it is therefore not surprising in our case to have just a singleton of the fused transcript out of 576 ESTs (transcript abundance 0.28%). This also demonstrates that to detect rare genetic processes operating in the venom gland, the library generated must be of high quality and that subsequent analyses must be performed very carefully. In turn, these analyses help elucidate in detail the principles of evolution of snake venom transcriptome which have led to the evolutionary success of the advanced snakes [128].

## Conclusion

The composition of snake venoms has been shown to be dependent on numerous factors, including phylogeny, diet, age, geography and even sex [129-132]. In general, greater similarity of venoms will be observed along broad phylogenetic lines (e.g., within-family than between-family). However, as this study has demonstrated, some toxins classically considered to occur in only one family, such as the 3FTxs, are actually broadly distributed among the advanced snakes (Colubroidea). The present capacity to detect low abundance toxins indicates a greater compositional similarity of venoms among advanced snakes than has been previously recognized. Further, we have demonstrated that in addition to gene duplication, exon shuffling or transcriptional splicing may also contribute to generating the diversity of toxins and toxin isoforms observed among snake venoms. Overall, the elucidation of the venom gland transcriptome of *S. c. edwardsii *contributes to a broader picture of toxin expression which complements and extends proteomic analysis of this venom [85]. These approaches can lead to the identification of new toxins and provides mechanistic explanations for their evolution and diversification. An unresolved question involves the relationship between the venom gland transcriptome and how this is ultimately translated to the final proteome. This variable proteomic composition in turn determines the complex and often difficult to resolve sequelae which frequently develop following envenomation by the different species of venomous snakes.

## Methods

### Venom extraction and collection of venom glands

Specimens of *Sistrurus c. edwardsii *(Desert Massasauga) were collected in Lincoln County, Colorado, USA under permits granted by the Colorado Division of Wildlife to SPM (permits #0456, 06HP456). Venom was extracted from adult snakes using standard manual methods [133]; venoms were then centrifuged to remove particulates, frozen and lyophilized. Prior to gland removal, snakes were extracted of venom. Four days later, when mRNA levels are presumed maximal [134], two snakes were anesthetized with isofluorane and then sacrificed by decapitation. Glands were then rapidly dissected from the snakes, cut into small pieces and placed in approximately 0.5 mL RNAlater (Qiagen) and frozen at -80°C until used.

### cDNA library construction and sequencing

Total RNA was extracted from a single venom gland using the RNeasy Mini Kit (Qiagen, Hilden, Germany). The integrity of total RNA was confirmed using agarose gel electrophoresis. The mRNA was purified using an mRNA isolation kit (Roche Applied Science, Mannheim, Germany). The purified total mRNA was used to make the cDNA library following the instructions of the SMART cDNA library construction kit (Vector used: λ TriplEx2) (Clontech, Mountain view, California, USA). Small size and incomplete cDNAs were removed by passing the library through CHROMA SPIN-400column. The library was packaged using Gigapack gold packaging extract (Stratagene, Cedar Creek, Texas, USA). Individual clones were rescued from randomly selected white plaques and grown in Luria broth + ampicillin medium. Plasmids were purified using the QIAprep spin miniprep kit (Qiagen, Hilden, Germany). Purified plasmids were sequenced by cycle sequencing reactions using the BigDye Terminator v3.1 kit (Applied Biosystem, Foster City, California, USA) and an automated DNA sequencer (Model 3100A, Applied Biosystem, Foster City, California, USA).

### RT-PCR

RT-PCR was performed in order to search for isoforms of 3FTx sequences in the venom gland. In brief, total RNA was isolated from venom glands as above and was used as template. The following primers were used for amplification: forward primer, 5' ATGAAAACTCTGCTGNTGATCCTGGNG 3' (N = A/C/G/T); reverse primer, 5' GGTTTATGGACCATCCTGTGGTAAAGGC 3'. Reverse transcription and subsequent amplification reactions were done using the one step RT-PCR protocol of Qiagen (Hilden, Germany). The amplified product was cloned into pDrive vector (Qiagen, Hilden, Germany) and 96 random clones were sequenced. RT-PCR was also performed to confirm the presence of fused toxin transcript in the venom using same procedure with the following primers: forward primer, 5' ATGTCTTCTGGAGGTCTTCTGCTG 3'; reverse primer, 5' TCCAG GACAGAAGAAGGCTCTGAT 3'.

### Bioinformatic analysis

Clustering of the ESTs was performed using the CAP3 program [135] after removing poor quality sequences and vector sequences using VecScreen from NCBI. We looked for Sfi I (A & B) recognition sequences in the ESTs and manually removed upstream and downstream sequences of these sites as well as poly(A) tails (at least 10 A's in a row) from the 3' ends. A minimum overlap of 50 bp and 100% identity in the overlap region were selected as criteria for the clustering. All clusters and singletons were subjected to BLAST searches (BLASTN and BLASTX as required) against the non-redundant database of NCBI (e-values cutoff < 10^-5 ^and having a good coverage of minimum 100 base pairs and >98% identity) for the putative identification of the genes [136]. Presence of signal peptides was predicted individually by submission of the sequences to the SignalP server as available in the Expasy website. Gene and protein alignments were done using the programs ClustalW and DNAMAN (Lynnon Corporation, Vaudreuil-Dorion, Quebec, Canada). The ratio between nonsynonymous (*d*_*N*_) and synonymous substitution (*d*_*S*_) were calculated using the SNAP program [137]. The program SNAP has been developed based on the method of [138] with incorporation of statistical analysis developed by [139].

### Phylogenetic analysis

Phylogenetic analysis was carried out using the program MEGA version 3.1[140], using Poisson-corrected distances, and trees were constructed applying bootstraps of 1000 replicates. PAUP 4.0b10 [102] was also used for Bootstrap, Neighbor Joining and Parsimony analyses. For the Bayesian inferences of phylogeny (based upon the posterior probability distribution of the trees: Markov chain Monte Carlo methods), MrBayes v3.1.2 [141] was used. The analysis was run for 5 × 10^6 ^generations in four chains and sampled every 100 generations, resulting in 50,000 sample trees. The log-likelihood score of each saved tree was plotted against the number of generations to determine the point at which the log-likelihood scores of the analysis reached the asymptote. The posterior probabilities for the clades were established by constructing a consensus tree of all trees generated after the completion of the burn-in phase.

We included the following sequences from three families of snakes in our analyses: the newly identified 3FTx sequences out of this study from *S. c. edwardsii *(Viperidae) [GenBank: DQ464281, DQ464282, DQ464283, DQ464284 and DQ464285]; *L. muta *(Viperidae) [GenBank: DY403363, DY403174], α-colubritoxin [Swiss-Prot: P83490] from *Coelognathus radiatus *(Colubridae), and non-conventional 3FTx sequences [Swiss-Prot: P81783, O42255 and P82935], 3FTx-like sequences [Swiss-Prot: Q02454, P62375, P24778, P24777, P24776, P01471, P62390, P01473, 229475, P01448, P01474, Q8UUK0 and Z54231] [142] and 3TFx sequences [Swiss-Prot: P10808, P01433, P01427, Q9YGC7, P10456, Q9YHV0, P80958, P60772, P34076, P01419 and P60237] from Elapidae. All these sequences belong to the short chain 3FTx family. A BLASTP search using [GenBank: DY403174] from *L. muta *venom found that a rat peptide sequence [GenBank: AAH63176] showed the highest homology to Ly6 antigen, which has been proposed to be a potential ancestor of snake venom 3FTxs [100,143], and this peptide was used as outgroup in our analysis.

## Accession numbers

Nucleotide sequence data reported here have been deposited in GenBank under accession numbers [GenBank: DQ464238–DQ464286]. ESTs are deposited in dbEST with accession numbers [dbEST: DY587747–DY588245 and DY625701–DY625710].

## List of abbreviations

EST, expressed sequence tag; ORF, open reading frame; PLA_2_, phospholipase A_2_; 3FTx, three-finger toxin; CRISP, cysteine-rich secretory protein; CLP, C-type lectins; BPP, bradykinin-potentiating peptides; CNP, C-type natriuretic peptides; LAO, L-amino acid oxidase; BPTI, bovine pancreatic trypsin inhibitor; VEGF, vascular endothelial growth factors; NGF, nerve growth factor; SVSP, snake venom serine proteinases; TLE, thrombin-like enzymes; CRD, cysteine-rich domain; NJ, neighbor-joining; TIC, transcription-induced chimerism.

## Authors' contributions

SP has designed and carried out experiments, analysed data, developed the concept and wrote the manuscript. SPM has collected the venom gland sample. RMK and SPM have edited the manuscript to improve its quality. SPM has helped in the phylogenetic analysis of 3FTxs sequences. All the authors have approved the final form of the manuscript.

## Supplementary Material

Additional file 1 It is a table showing the distribution of snake venom toxin families among Superfamily Colubroidae. Click here for file

Additional file 2 It is a table showing the clusters of ESTs, number of clones in each cluster and their putative identity. Click here for file

Additional file 3 It is a table showing the clusters of ESTs encoding cellular proteins. Click here for file

Additional file 4 ClustalW alignment between 12S ribosomal RNA sequence DQ464268 (from this study) and AF057227 (used for taxonomic identification of *S. c. edwardsii*). Polyadenylation signal sequence is underlined. Click here for file

Additional file 5 ClustalW alignment of PIII metalloproteinases (only proteinase domain is shown). Cysteine residues which are conserved are marked in grey and variable in black. Accession numbers of the used sequences are as follows: VAP1 [GenBank: BAB18307], HV1 [GenBank: BAB60682], Halysase [GenBank: 27465044], VLAIP-A [GenBank: 61104775], VLAIP-B [GenBank: 61104777], Kaouthiagin [Swiss-Prot: P82942], Berythractivase [Swiss-Prot: Q8UVG0], Ecarin [Swiss-Prot: Q90495], Jararhagin [Swiss-Prot: P30431], Bothropasin [Swiss-Prot: O93523], Acurhagin [Swiss-Prot: Q6Q274], Catrocollastatin [Swiss-Prot: Q90282], Atrolysin [Swiss-Prot: Q92043], Stejnihagin-A [Swiss-Prot: Q3HTN1], Stejnihagin-B [Swiss-Prot: Q3HTN2], HR1A [Swiss-Prot: Q8JIR2], HR1B [Swiss-Prot: P20164], HF3 [GenBank: 31742525]. P, signal peptide domain; PRO, pro-domain; S, spacer; DISIN, disintegrin domain; CRD, cysteine-rich domain Click here for file

Additional file 6 Bayesian tree generated from 39 aligned 3FTx sequences as described in Materials and Methods. Numbers on branches indicate percentage of posterior clade probability. 3FTx sequences from *S. c. edwardsii *and *L. muta *libraries are marked with a filled circle and triangle respectively. Click here for file
