# Supplementary material for: The venom gland transcriptome of the Desert Massasauga Rattlesnake (Sistrurus catenatus edwardsii): towards an understanding of venom composition among advanced snakes (Superfamily Colubroidea)
Source: BMC Mol Biol. 2007 Dec 20;8:115. doi: 10.1186/1471-2199-8-115 (PMC2242803; doi:10.1186/1471-2199-8-115)
Supplement: Additional file 1 — It is a table showing the distribution of snake venom toxin families among Superfamily Colubroidae. [file 1471-2199-8-115-S1.pdf]

COLUBROIDEA

	Colubridae	Elapidae	Atractaspididae	Viperidae
**ENZYMATIC**				
Metalloproteinase	+	+	+	+
PLA_2	+	+		+
LAO	+	+		+
Serine proteinase	+			+
Serine proteinase related to factor Xa		⊕		
Phosphodiesterase	+	+		+*
Acetylcholineesterase	+	+		
Hyaluronidase		⊕		
Dipeptidyl peptidase				⊕
**NON-ENZYMATIC**				
Sarafotoxin			⊕	
Disintegrin				⊕
VEGF				⊕
NGF		+		+
3FTx	+	+		+*
CLP	+	+		+
CRISP	+	+		+
BPP-CNP	+	+		+
Kunitz/BPTI		+		+
Vespryns		+		+
Cobra venom factor		⊕		
AVIT peptide		⊕		
Waprins		⊕		
Cystatin				⊕
Crotamine				⊕
Waglerin				⊕
Novel toxin (Kunitz/BPTI + Waprins)				⊕*

This table has been compiled collecting the reports for the presence of either gene sequence encoding toxin or pharmacological activity in the venom. Positive circles mark the presence of a family of toxin to a particular snake family. Asterisk marks report the interesting family of toxins which are identified from this work.
