# Supplementary material for: The venom gland transcriptome of the Desert Massasauga Rattlesnake (Sistrurus catenatus edwardsii): towards an understanding of venom composition among advanced snakes (Superfamily Colubroidea)
Source: BMC Mol Biol. 2007 Dec 20;8:115. doi: 10.1186/1471-2199-8-115 (PMC2242803; doi:10.1186/1471-2199-8-115)
Supplement: Additional file 2 — It is a table showing the clusters of ESTs, number of clones in each cluster and their putative identity. [file 1471-2199-8-115-S2.pdf]

Cluster	Number of clones	Putative identity
**Serine proteinases**		
SCESP1	31	Calobin precursor
SCESP2	27	Catroxase I precursor
SCESP3	15	Catroxase II precursor
SCESP4	13	Serine proteinase 3 precursor
SCESP5	11	Acubin 2 precursor
SCESP6	06	Gyroxin-like B2.1 precursor
SCESP7	06	Salmonase precursor
SCESP8	06	Venom serine protease 5 precursor
SCESP9	05	Venom serine protease 1 precursor
SCESP10	03	Preprotrimubin mRNA precursor
SCESP11	02	Serine protease KN9 precursor
SCESP12	02	Hypothetical serine protease
SCE144	01	*Trimeresurus gramineus* mRNA for serine protease
SCE343	01	Serine protease CL4 precursor
SCE408	01	Putative serine proteinase (sp1gene)
SCE395	01	Serine protease KN7 precursor
SCE435	01	*Trimeresurus flavoviridis* serine protease
SCE488	01	*Gloydius saxatilis* thrombin-like enzyme
**Metalloproteinases and Disintegrins**		
SCEMP1	22	*Agkistrodon metalloproteinase*-disintegrin-like protein
SCEMP2	04	Hemorrhagic metalloproteinase HR1a precursor
SCEMP3	03	Hemorrhagic metalloproteinase HF3 precursor
SCEMP4	03	Atrolysin a precursor
SCEMP5	02	*Bothrops jararacussu* BOJUMET II precursor
SCEMP6	02	*Agkistrodon contortrix contortrix* acostatin beta chain
SCE456	01	Vascular apoptosis-inducing protein 1 precursor
SCE062	01	Insularinase and insularin precursor
SCE284	01	*Agkistrodon contortrix* metalloproteinase precursor
SCE389	01	*Gloydius saxatilis* metalloproteinase precursor
SCE409	01	*Bothrops insularis* metalloproteinase precursor
SCE422	01	*Vipera stejnegeri* stejnihagin-A
SCE425	01	*Gloydius halys* metalloprotease (Mt-a) precursor
SCE157	01	*Trimeresurus flavoviridis* metalloproteinase
**Phospholipase A_2**		
SCEPLA$_2$	84	*Sistrurus catenatus* tergeminus PLA$_2$ precursor
SCE039	01	*Bothrops insularis* cluster BITP01A PLA$_2$ precursor
SCE070	01	*Crotalus viridis viridis* strain E6e PLA$_2$ precursor
SCE117	01	Snake BP-II gene for phospholipase A_2
SCE135	01	*Trimeresurus gramineus* gTgPLA6a gene for PLA$_2$
SCE295	01	*Trimeresurus stejnegeri* PLA$_2$ isozyme Ts-A3 mRNA
SCE367	01	*Crotalus durissus terrificus* mRNA for crotoxin A
SCE376	01	*Trimeresurus stejnegeri* PLA$_2$ isozyme Ts-A3 mRNA
SCE403	01	*Crotalus viridis viridis* strain E6e PLA$_2$ precursor
SCE404	01	*Trimeresurus stejnegeri* PLA$_2$ isozyme Ts-A3 mRNA
SCE405	01	*Bothrops insularis* cluster BITP01A PLA$_2$ precursor
SCE417	01	*Crotalus durissus terrificus* mRNA for crotoxin A
SCE445	01	*Bothrops jararacussu* myotoxic A_2-like PLA$_2$ mRNA
SCE235	01	Snake pgPLA2a gene for phospholipase A_2
SCE241	01	*Bothrops jararacussu* myotoxic A2-like PLA$_2$ mRNA
SCE411	01	*Crotalus durissus terrificus* crotoxin, CB subunit

**Phosphodiesterase**		
SCE269	01	*Mus musculus* ectonucleotide phosphodiesterase 3
**L-Amino acid oxidase**		
SCELAO	13	L-amino acid oxidase precursor
**C-type lectins**		
SCECLP1	03	*Agkistrodon blomhoffi* mamushigin beta
SCE049	01	*Trimeresurus flavoviridis* factor IX/factor X binding protein A chain
SCE328	01	*Trimeresurus stejnegeri* stejaggregin-B beta chain-1 mRNA
**Growth factors**		
SCEGF1	17	*Bothrops insularis* VEGF precursor
SCE302	01	*Protobothrops mucrosquamatus* VEGF precursor
SCE373	01	*Bothrops jararaca* VEGF precursor
SCE419	01	*Trimeresurus flavoviridis* VEGFprecursor
SCE468	01	*Protobothrops mucrosquamatus* VEGF precursor
SCE467	01	*Crotalus durissus terrificus* NGF precursor
SCE339	01	similar to connective tissue growth factor *Gallus gallus*
**CRISP**		
SCECRISP1	20	*Crotalus atrox* catrin mRNA
SCE052	01	*Trimeresurus jerdonii* cysteine-rich venom protein
SCE322	01	*Agkistrodon piscivorus piscivorus* piscivorin mRNA
SCE352	01	*Agkistrodon blomhoffi* ablomin mRNA
SCE391	01	*Trimeresurus jerdonii* cysteine-rich venom protein
SCE407	01	*Trimeresurus mucrosquamatus* cysteine-rich protein
SCE431	01	*Trimeresurus flavoviridis* triflin mRNA
SCE268	01	*Agkistrodon blomhoffi* ablomin mRNA
SCE394	01	*Trimeresurus jerdonii* cysteine-rich venom protein
**Bradykinin-potentiating peptides and C-type natriuretic peptides**		
SCE492	01	BPP and CNP precursor
**Three finger toxin**		
SCE164	01	*Naja atra* gene for cardiotoxin 10
SCE215	01	*Bungarus multicinctus* mRNA for neurotoxin
SCE390	01	*Naja naja atra* mRNA for cobrotoxin homolog
**Novel toxin**		
SCE290	01	*Vipera ammodytes* trypsin inhibitor preproprotein
**Iron binding protein**		
SCEHYPO1	04	*Lamprophis fuliginosus* mRNA for iron binding protein
