# Supplementary material for: The venom gland transcriptome of the Desert Massasauga Rattlesnake (Sistrurus catenatus edwardsii): towards an understanding of venom composition among advanced snakes (Superfamily Colubroidea)
Source: BMC Mol Biol. 2007 Dec 20;8:115. doi: 10.1186/1471-2199-8-115 (PMC2242803; doi:10.1186/1471-2199-8-115)
Supplement: Additional file 3 — It is a table showing the clusters of ESTs encoding cellular proteins. [file 1471-2199-8-115-S3.pdf]

Cluster	Number of clones	Putative identity
**General metabolism**		
SCE008	01	Glycerol-3-phosphate dehydrogenase 1
SCE014	01	Glyoxylase 1
SCE055	01	Cytochrome oxidase subunit VIII(H)
SCE063	01	Cytochrome P450 1A5
SCE100	01	Cytochrome c oxidase subunit VIIb precursor
SCE121	01	Cytochrome C oxidase copper chaperone
SCE178	01	Phosphoribosylaminoimidazole carboxylase
SCE154	01	Similar to V-ATPase C2 subunit
SCE427	01	S-adenosylmethionine decarboxylase 1
SCE491	01	Short chain dehydrogenase
SCE264	01	Similar to NADH dehydrogenase (ubiquinone) 1
SCE092	01	ATP synthase beta chain
SCE452	01	Ferritin
**Transcription and translation apparatus**		
SCERP1	02	40S ribosomal protein S4
SCERP2	02	40S ribosomal protein SA (p40) (laminin receptor)
SCE500	01	Ribosomal protein L36a isoform 1
SCE501	01	Ribosomal protein S29 isoform 1
SCE502	01	Ribosomal protein L37
SCE503	01	Ribosomal protein S26
SCE504	01	Ribosomal protein L23
SCE505	01	Ribosomal protein S27a
SCE506	01	Ribosomal protein L19 variant
SCE507	01	40S ribosomal protein S14
SCE508	01	Ribosomal protein L34
SCE509	01	Mitochondrial ribosomal protein L34
SCE005	01	Translation initiation factor 2
SCE243	01	Translation initiation factor 1
SCE228	01	Elongation factor TU
SCE300	01	TATA-box binding protein
SCE163	01	Elongation factor Tu GTP binding domain
SCE291	01	U3 small nucleolar ribonucleoprotein
SCE386	01	Ribonuclease P/MRP subunit, precursor 5
**Post-translational processing and sorting**		
SCEHYPO2	02	Signal sequence receptor
SCEHYPO4	02	Calreticulin
SCEHYPO7	02	Transport protein Sec61 beta subunit
SCE139	01	Similar to Protein disulfide isomerase A6 precursor
SCE377	01	*Oxyuranus scutellatus scutellatus* PDI
SCE015	01	Thioredoxin domain containing protein 1 precursor
SCE438	01	Calcium and integrin binding 1
SCE003	01	YME1-like 1 protease
SCE131	01	Putative transporter
SCE388	01	Glutathione transferase zeta 1
SCE470	01	Glutathione S-transferase theta 1
**Protein Modification and Degradation**		
SCE030	01	Proteasome maturation factor UMP1
SCE073	01	Ubiquitin A-52 residue ribosomal protein
SCE223	01	Proteasome activator rPA28 subunit beta
SCE317	01	Polyubiquitin
**Structural proteins**		
SCE200	01	Torsin A
SCE185	01	Actin filament, gelsolin-like
SCE311	01	Cytokeratin 19

SCE444	01	Alpha-tubulin
**Cell regulation and other functions**		
SCEHYPO3	03	Similar to Hypothetical protein FLJ22662 of Gallus gallus
SCEHYPO5	02	Thymosin beta variant
SCEHYPO6	03	Immunoglobulin heavy chain
SCE179	01	Immunoglobulin M constant region
SCE366	01	Peptidylprolyl isomerase
SCE064	01	Similar to DnaJ homolog
SCE050	01	Cyclophilin A
SCE041	01	Putative thrombin inhibitor
SCE384	01	Similar to multiple coagulation factor deficiency 2
SCE078	01	Chemokine precursor
SCE165	01	Lens fiber membrane intrinsic protein
SCE339	01	Connective tissue growth factor-like protein
SCE356	01	Poliovirus receptor-related protein
SCE021	01	Serine (or cysteine) proteinase inhibitor
SCE060	01	Similar to Copine III
SCE068	01	Similar to MGC89718 protein (Xenopus tropicalis)
SCE079	01	ATP/ADP antiporter
SCE111	01	Predicted to similar to 14 kDa phosphohistidine phosphatase
SCE086	01	Parvalbumin beta
SCE091	01	Similar to human neuronal protein
SCE98	01	Similar to small EDRK-rich factor 1
SCE102	01	Mak3p homolog
SCE112	01	Adaptor protein complex AP-1
SCE129	01	Similar to tRNA selenocysteine associated protein
SCE167	01	Similar to tRNA selenocysteine associated protein isoform 1
SCE175	01	Insulin-induced protein
SCE183	01	Nucleic acid binding protein 2
SCE188	01	Similar to prion protein interacting protein 1
SCE193	01	Clathrin
SCE209	01	Clathrin adaptor complex small chain
SCE245	01	Apoptotic suppressor
SCE227	01	GTP binding protein
SCE279	01	Rab subfamily of small GTPases
SCE297	01	Benzodiazepine receptor
SCE349	01	Similar to basic leucine zipper and W2 domains 1 isoform3
SCE350	01	Progesterone receptor membrane component 2
SCE357	01	Similar to sulfotransferase 1B
SCE398	01	Rho (Ras homology) subfamily of Ras-like small GTPases
SCE399	01	Golgi transport 1 homolog B
SCE441	01	Eukaryotic porin
SCE460	01	Fibroblast growth factor binding protein
SCE471	01	SH3 domain-containing protein 1A
SCE125	01	ATP synthase D chain
SCE187	01	Calbindin
SCE072	01	B-cell translocation/anti-proliferative gene
**Hypothetical sequences**		
SCE387	01	Hypothetical/no match
SCE027	01	Hypothetical-similar to FLJ00261 protein
SCE037	01	Hypothetical-unnamed protein product
SCE042	01	Hypothetical protein TP02_0326
SC110	01	Hypothetical protein product -*Paramecium tetraurelia*
SCE120	01	Hypothetical SpoVR-like family protein
SCE123	01	Hypothetical protein -*Xenopus tropicalis*
SCE189	01	Hypothetical-no significant similarity found
SCE191	01	Hypothetical-no significant similarity found

SCE190	01	Hypothetical protein-no significant similarity found
SCE226	01	Hypothetical peroxiredoxin 6 like protein
SCE277	01	Hypothetical-no significant similarity found
SCE299	01	Hypothetical-no significant similarity found
SCE308	01	Hypothetical Leucine Rich Repeat family protein
SCE313	01	Hypothetical nitrous-oxide reductase
SCE354	01	Hypothetical protein for -*Xenopus laevis*
SCE358	01	Hypothetical KIAA0491-*Homo sapiens*
SCE393	01	Hypothetical-no significant similarity found.
SCE397	01	Hypothetical (XAP-5 protein)-*Rattus norvegicus*
SCE437	01	Hypothetical protein
SCE265	01	Hypothetical-no significant similarity found
SCE269	01	Hypothetical-no significant similarity found.
SCE318	01	Hypothetical protein pEA28_0-*Erwinia amylovora*
SCE365	01	Hypothetical to ribosomal protein S14-*Homo sapiens*
SCE 137	01	Hypothetical protein OsJ_008985-*Oryza sativa*
SCE147	01	Hypothetical IgM heavy chain like
SCE081	01	Hypothetical-no significant similarity found
SCE053	01	Hypothetical-no significant similarity found.
SCE051	01	Hypothetical-no significant similarity found
SCE136	01	Hypothetical-no significant similarity found
SCE182	01	Hypothetical proline/serine-rich coiled-coil 2-*Mus musculus*
SCE218	01	Hypothetical-*Homo sapiens* insulin induced gene 1
SCE237	01	Hypothetical solute carrier family 30
SCE246	01	Hypothetical-no significant similarity found
SCE250	01	Hypothetical hCG2036582, isoform CRA_b-*Homo sapiens*
SCE251	01	Hypothetical-no significant similarity found
SCE252	01	Hypothetical-no significant similarity found
SCE273	01	Hypothetical protein for MGC:80952)-*Xenopus laevis*
SCE414	01	Hypothetical-no significant similarity found
SCE424	01	Hypothetical-no significant similarity found
SCE494	01	Hypothetical similar to ribosomal protein L32 isoform 2
SCE402	01	Hypothetical-no significant similarity found.
