# Supplementary material for: The venom gland transcriptome of the Desert Massasauga Rattlesnake (Sistrurus catenatus edwardsii): towards an understanding of venom composition among advanced snakes (Superfamily Colubroidea)
Source: BMC Mol Biol. 2007 Dec 20;8:115. doi: 10.1186/1471-2199-8-115 (PMC2242803; doi:10.1186/1471-2199-8-115)
Supplement: Additional file 4 — ClustalW alignment between 12S ribosomal RNA sequence DQ464268 (from this study) and AF057227 (used for taxonomic identification of S. c. edwardsii). Polyadenylation signal sequence is underlined. [file 1471-2199-8-115-S4.pdf]

```
DQ464268  GCCTAGCCGTAAAATACAATTAAACTACCAATTGTCCGCCAAACAACTACGAGTACTACTTAAAAC
AF057227  GCCTAGCCGTAAAATACAATTAAACTACCAATTGTCCGCCAAACAACTACGAGTACTACTTAAAAC
          *****************************************************************

DQ464268  TTAAAAGACTTGACGGTGCTTCACCACGCCCTAGAGGAGCCTGTCTAGCAACCGATAATCCACGAT
AF057227  TTAAAAGACTTGACGGTGCTTCACCACGCCCTAGAGGAGCCTGTCTAGCAACCGATAATCCACGAT
          *****************************************************************

DQ464268  TAACCCAGCCCCCCCTAGCCCAACAGTCTATATACCGCCGTCGCCAGCTTACCTTGTAAAAGAAAT
AF057227  TAACCCAGCCCCCCCTAGCCCAACAGTCTATATACCGCCGTCGCCAGCTTACCTTGTAAAAGAAAT
          *****************************************************************

DQ464268  AAAGTAAGCCAAACAGTAATTTCAACTAAAACGACAGGTCGAGGTGTAACTAATGAGGGGGACTAA
AF057227  AAAGTAAGCCAAACAGTAATTTCAACTAAAACGACAGGTCGAGGTGTAACTAATGAGGGGGACTAA
          *****************************************************************

DQ464268  GATGGGCTACATTCTCCTGACTGAGGATACGAACAATACTATGAAATTAGTATTTAAAGGCGGATT
AF057227  GATGGGCTACATTCTCCTGACTGAGGATACGAACAATACTATGAAATTAGTATTTAAAGGCGGATT
          *****************************************************************

DQ464268  TAGCAGTAAGATAAGAATAAAATACTTAACTGAACATAACGCAATGAAGCGCGTACACACCGCCCG
AF057227  TAGCAGTAAGATAAGAATAAAATACTTAACTGAACATAACGCAATGAAGCGCGTACACACCGCCCG
          *****************************************************************

DQ464268  TCACCCCTGCTAACACAACACATAGTTAAATAAACAACCCAAAAAAAAAAAAAAAAAAAAAAAAAA
AF057227  TCACCCCTGCTAACAC--------------------------------------------------
          ****************
```
