# Supplementary material for: The venom gland transcriptome of the Desert Massasauga Rattlesnake (Sistrurus catenatus edwardsii): towards an understanding of venom composition among advanced snakes (Superfamily Colubroidea)
Source: BMC Mol Biol. 2007 Dec 20;8:115. doi: 10.1186/1471-2199-8-115 (PMC2242803; doi:10.1186/1471-2199-8-115)
Supplement: Additional file 5 — ClustalW alignment of PIII metalloproteinases (only proteinase domain is shown). Cysteine residues which are conserved are marked in grey and variable in black. Accession numbers of the used sequences are as follows: VAP1 [GenBank: BAB18307], HV1 [GenBank: BAB60682], Halysase [GenBank: 27465044], VLAIP-A [GenBank: 61104775], VLAIP-B [GenBank: 61104777], Kaouthiagin [Swiss-Prot: P82942], Berythractivase [Swiss-Prot: Q8UVG0], Ecarin [Swiss-Prot: Q90495], Jararhagin [Swiss-Prot: P30431], Bothropasin [Swiss-Prot: O93523], Acurhagin [Swiss-Prot: Q6Q274], Catrocollastatin [Swiss-Prot: Q90282], Atrolysin [Swiss-Prot: Q92043], Stejnihagin-A [Swiss-Prot: Q3HTN1], Stejnihagin-B [Swiss-Prot: Q3HTN2], HR1A [Swiss-Prot: Q8JIR2], HR1B [Swiss-Prot: P20164], HF3 [GenBank: 31742525]. P, signal peptide domain; PRO, pro-domain; S, spacer; DISIN, disintegrin domain; CRD, cysteine-rich domain [file 1471-2199-8-115-S5.pdf]

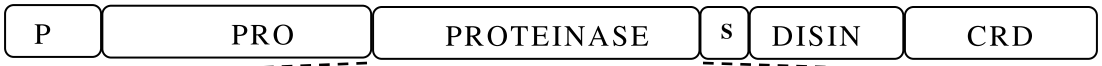


```
                   11                                                                                     103     111
DQ464249           LDAKKYVEFVVVLDHGMYTKYKDDLDKIKTRIYEIVNTMNEIYIPLNIRVALVRLEIWSNGDLINVSSAANVTLDSFRNWRVTDLLRRKSHDNAQLLTTTD
Jararhagin         -DPYKYIEFFVVVDQGTVTKNNGDLDKIKARMYELANIVNEIFRYLYMHVALVGLEIWSNGDKITVKPDVDYTLNSFAEWRKTDLLTRKKHDNAQLLTAID   PIIIa
Bothropasin        -NPFRYVELFIVVDQGMVTKNNGDLDKIKARMYELANIVNEILRYLYMHAALVGLEIWSNGDKITVKPDVDYTLNSFAEWRKTDLLTRKKHDNAQLLTAID
Acurhagin          -DPFKYVETVVVVDKAMVTKYNGDLDKIKTKMYEAANNMNEMYRYMFFRVVMVGLIIWTEEDKITVKPDVDYTLNAFAEWRKTYLLAEKKHDNAQLITGID
Catrocollastatin   -NPFRFVELFLVVDKAMVTKNNGDLDKIKTRMYEIVNTVNEIYRYMYIHVALVGLEIWSNEDKITVKPEAGYTLNAFGEWRKTDLLTRKKHDNAQLLTAID
DQ464255           LNAKKYVKLFLVADYVMYLKYGRNLTAVRTRMYDTVNVITPIYHRMNIHVALVGLEIWSNRDKIIVQSSPDVTLDLFAKWRATDLLRRKSHDNAQLLTGIN
VAP1               LNAKKYVKLFLVADYIMYLKYGRNLTAVRTRMYDIVNVITPIYHRMNIHVALVGLEIWSNTDKIIVQSSADVTLDLFAKWRATDLLSRKSHDNAQLLTGIN
HV1                LNAKKYVKFFLVADHIMYLKYGRNLTTLRTRMFDTVNIVNQILQRINIHVALIGIEIWSKEDKIIVQSVPDVTLKLFATWRESVLLKRKNHDNAHLLTGIN   PIIIb
Halysase           LNAKKYVKLVMVADYIMYLKYDRNLTTVRTRMYDIVNVINVIYQRMNIHVALVGLEIWSNKDKFILRSAADVTLKLFATWRETDLLKRKSHDNAQLLTGIN
VLAIP-A            LNSPKYIKLVIVADYIMFLKYGRSLITIRTRIYEIVNILNVIYRVLNIYIALLGLEIWNNGDKINVLPETKVTLDLFGKWRERDLLNRRKHDNAQLLTDIN
VLAIP-B            LKSKKYVELVIVADYIMFWKYDRSLSTIRTRIYEIVNTLNVIYRFLNIYIALVAVEIWSKGDLINVTSSAYDTLDSFGEWRERDLLNRKRHDNAQLLTGIN
Stejnihagin-A      --PRRYVKLAIVADRRMYMKHQKNLK---PWVFQMVNSVHQIYRSMNVLIALVYLNIWKKNDKITVQSASDVTLDLFAEWRETVLLRRKKHDCAHLLTAID
Stejnihagin-B      --PRRYVKLAIVADHRMYTKHKKNLK---PWVFQMVNSVHQIYRSMNVLIALVYLNIWKKNDKITAQSASNVTLDLFGNWRETVLLKRKRHDCAQLLTAID   PIIIc
HR1B               --PRRYIKLAIVVDHGIVTKHHGNLKKIRKWIYQLVNTINNIYRSLNILVALVYLEIWSKQNKITVQSASNVTLDLFGDWRESVLLKQRSHDCAQLLTTID
DQ464251           --TKRYVELVIVADHGMFMKHN---ETLRTWVFQMVNTINEIYIPLNIRFALVGLEIWSNGDKITVQSSAHNTLHLFGNWRKRVLLKRKKHDNAQLLTSID
DQ464250           LNNFRYIELVIVADYRMFTKYNRKLTEVKTWVYEIVNTLNEIYRYLYIRVALVGLEIWSNGDLSNVTLSADDTLDSFGEWRKRDLLRRKSHGNAQLLTAID   PIII0
Kaouthiagin        LQAEKYIEFYVIVDNRMYRYYNYDKPAIKIRVYEMINAVNTKFRPLKIHIALIGLEIWSNEDKFEVKPAASVTLKSFREWRQTVLLPRKRNDNAQLLTGIN
HF3                -NHYKYIELVILADYRMVTKNNGDLGKIRTKIYEIVNILNEIFRYLYIRIALVGIEIWSNADLSNVTLSADDTLASFGTWRGTVLLKRKSHDNAQLLTAID
Berythractivase    LDAKKYVEFVVVLDHGMYKKYKDDLDKIKRRIYEIVNTMNEMFIPLNICVALTGLEIWSKGDKINVTSESWFTLILFTNWRGADLLKRKSHDNAQLLTNTD
Ecarin             FE-KKFIELVVVVDHSMVTKYNNDSTAIRTWIYEMLNTVNEIYLPFNIRVALVGLEFWCNGDLINVTSTADDTLHSFGEWRASDLLNRKRHDHAQLLTNVT
Atrolysin          --TKRYVELVIVADHRMFTKYNGNLKKIRKWIYQIVNTINEIYIPLNIRVALVRLEIWSNGDLIDVTSAANVTLKSFGNWRVTNLLRRKSHDNAQLLTAID
HR1A               LNNFRFIELVIVADYRMFTKFNSNLNEVKTWVYEIVNTLNEIYRYLYVRVALVALEVWSNGDLSSVTLSAYDTLDSFGEWRKRDLLKRKSHDNAQLLTAID
                   112                                                         181           195              211
DQ464249           FDGDTVGLAYMRSMCQPRGSVGVIQEHSTINLLMAVTMAHEMGHNLGMSHDGNQCHCGAPSCIMADRLSHQPSTQFSDCSEKYCRTYLINSRPQCILNEP
Jararhagin         FNGPTIGYAYIGSMCHPKRSVGIVQDYSPINLVVAVIMAHEMGHNLGIHHDTGSCSCGDYPCIMGPTISNEPSKFFSNCSYIQCWDFIMNHNPECIINEP   PIIIa
Bothropasin        FNGPTIGYAYIGSMCHPKRSVAIVEDYSPINLVVAVIMAHEMGHNLGIHHDTDFCSCGDYPCIMGPTISNEPSKFFSNCSYIQCWDFIMKENPQCILNEP
Acurhagin          FRGSIIGYAYIGSMCHPKRSVGIIQDYSPINLVLAVIMAHEMGHNLGIHHDDGYCYCGGYPCIMGPSISPEPSKFFSNCSYIQCWDFIMNHNPECIDNEP
Catrocollastatin   LDR-VIGLAYVGSMCHPKRSTGIIQDYSEINLVVAVIMAHEMGHNLGINHDSGYCSCGDYACIMRPEISPEPSTFFSNCSYFECWDFIMNHNPECILNEP
DQ464255           FNGPTAGLAYLGGICNTMYSAGIVQDHSKIHHLVAIAMAHEMGHNLGMDHDKETCTCGTRPCVMAGTLSCEASFLFSDCSQKEHREFLIKNMPQCILKKP
VAP1               FNGPTAGLGYLGGICNTMYSAGIVQDHSKIHHLVAIAMAHEMGHNLGMDHDKDTCTCGTRPCVMAGALSCEASFLFSDCSQKDHREFLIKNMPQCILKKP
HV1                FNGPTAGLAYLGGICKPMYSAGIVQDHNKIHHLVAIAMAHEMGHNLGMDHDKDTCTCRAKACVMAGTLSCDASYLFSDCSRQEHRAFLIKNMPQCILKKP   PIIIb
Halysase           FNGPTAGLGYLGGICNPMYSAGIVQDHNKIHHLVAIAMAHEMGHNLGIDHDKDTCTCGAKSCVMAGTLSCEASYLFSDCSRKEHQAFLIKDMPQCILKKP
VLAIP-A            FNGPTAGLGYVGSMCDPQYSAGIVQDHNKVNFLVALAMAHEMGHNLGMEHDEIHCTCGAKSCIMSGTLSCEASIRFSNCSREEHQKYLINKMPQCILNKP
VLAIP-B            FNGPSAGRGFVGRMCQPKYSVGIVQDHSKIYLLVASAMAHEMGHNLGMDHDRIDCTCGAKSCIMSGILRCETSYLFSDCSREEHRKYLINKMPQCILNKP
Stejnihagin-A      FDGPTIGRAHIASMCNSKLSVGIVQNYTEINLVNAIVMAHELGHNLGISHDGNQCNC--HTCIMSAVISNPPSERFSNCSEDYHQSFLTAYNPQCILNAP
Stejnihagin-B      FDGPTIGRAHVSSVCDPKRSTGIVQNYTEINLVNAVIMAHELGHNLGMDHDGNQCNC--HACIMSAVINNPPSERFSGCSMGYYQTFLTAYNPQCILNAL   PIIIc
HR1B               FDGPTIGKAYTASMCDPKRSVGIVQDYSPINLVVAVIMTHEMGHNLGIPHDGNSCTCGGFPCIMSPMISDPPSELFSNCSKAYYQTFLTDHKPQCILNAP
DQ464251           FDGRTVGLAHVSSMCNQKLSTGVIQDHSAINLVMAVTMAHELGHNLGMDHDIKYCTCGAPACVMGNTLSEQPSYGFSNCSQNQYQTFIINYNPQCILNEP
DQ464250           FNGTTIGLAHVASMCELKRSTGIVQDHSPINLLVAVTMAHEMGHNLGIRHDKKYCTCGGYSCIMSAVLSHQASKYFSNCSYNQYWNYINFYKPQCILNEP   PIII0
Kaouthiagin        LNGTAVGIAYPGSLCT-QRSVFVVQDYNRRMSLVASTMTHELGHNLGIHHDEASCICIPGPCIMLKKRTAPA-FQFSSCSIRDYQEYLLRDRPQCILNKP
HF3                FDGQTIGIANIASMCNQNKSVGVVMDYSPINLVVAVIMAHEMGHNLGINHDTGSCSCGGYSCIMAPEISDQPSKLFSNCSKQAYQRYINYYKPQCILNEP
Berythractivase    FDGSTIGRAHIGSMCHPYLSVGIIQDYSPVNLLVASTMAHEMGHNLGMHHDNDTCTCGAPSCVMAAAISKDPSKLFSNCSQEYQRKYLIKNRPQCLLNKP
Ecarin             LDHSTLGITFVYGMCKSDRSVELILDYSNITFNMAYIIAHEMGHSLGMLHDTKFCTCGAKPCIMFGKESIPPPKEFSSCSYDQYNKYLLKYNPKCILDPP
Atrolysin          LDEETLGLAPLGTMCDPKLSIGIVQDHSPINLLVAVTMAHELGHNLGMVHDENRCHCSTPACVMCAVLRQRPSYEFSDCSLNHYRTFIINYNPQCILNEP
HR1A               FNGTIIGLAHVASMCDPKCSTGIVQDYSSRNLVVAVIMAHEMGHNLGIRHDRENCTCHANSCIMSAVISDQPSKYFSNCSHVQYWNYINDDEPQCILNEP
```
