# Supplementary figures and images for: The venom gland transcriptome of the Desert Massasauga Rattlesnake (Sistrurus catenatus edwardsii): towards an understanding of venom composition among advanced snakes (Superfamily Colubroidea)
Source: BMC Mol Biol. 2007 Dec 20;8:115. doi: 10.1186/1471-2199-8-115 (PMC2242803; doi:10.1186/1471-2199-8-115)

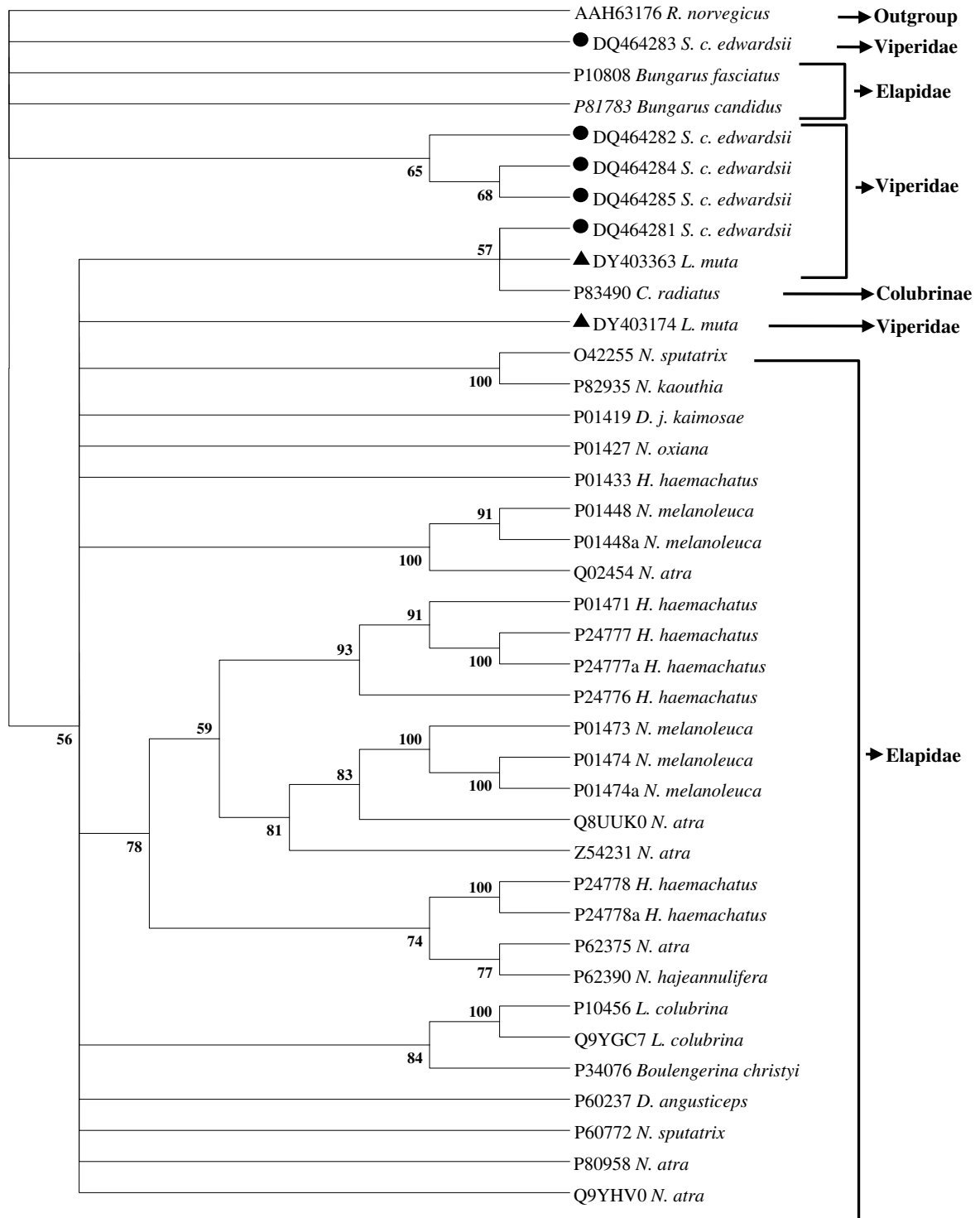

Supplement: Additional file 6 — Bayesian tree generated from 39 aligned 3FTx sequences as described in Materials and Methods. Numbers on branches indicate percentage of posterior clade probability. 3FTx sequences from S. c. edwardsii and L. muta libraries are marked with a filled circle and triangle respectively. [file 1471-2199-8-115-S6.pdf]
